# Short-term effects of Ahmed valve implantation on ocular biometry and corneal biomechanics in neovascular glaucoma

**DOI:** 10.1186/s13104-025-07313-0

**Published:** 2025-07-01

**Authors:** Ali Azimi, Alireza Attar, Aidin Meshksar, Hooman Razmi, Mohammad Hassan Jalalpour, Amirmohammad Fathian, Adele Yarmohammadi

**Affiliations:** 1 Department of Ophthalmology, Poostchi Ophthalmology Research Center, School of Medicine, Shiraz University of Medical Sciences, Shiraz, Iran; 2 UC San Diego Shiley Eye Institute, San Diego, USA

**Keywords:** Ahmed glaucoma valve implantation, Neovascular glaucoma, Corneal biomechanical

## Abstract

**Objective:**

To evaluate the impact of Ahmed glaucoma valve (AGV) implantation on ocular biometric and corneal biomechanical parameters in patients with neovascular glaucoma (NVG), with the goal of understanding how these changes influence intraocular pressure (IOP) control, surgical outcomes, and long-term prognosis.

**Results:**

Eighteen NVG patients (mean age: 57.44 ± 10.33 years) underwent AGV implantation. A significant reduction in IOP was observed, from 36.65 ± 12.3 mmHg preoperatively to 15.5 ± 2.64 mmHg postoperatively (p < 0.001). Axial length decreased from 23.42 ± 0.76 mm to 23.21 ± 0.76 mm (p < 0.001), while anterior chamber depth increased significantly (p = 0.037). Both corneal keratometry and central corneal thickness showed significant postoperative decreases (p < 0.001). Corneal biomechanical parameters such as peak distance and deformation amplitude increased significantly (p = 0.004 and p = 0.001, respectively), whereas no significant changes were noted in applanation lengths and velocities (p > 0.05). These findings suggest that AGV implantation leads to substantial alterations in ocular structure and biomechanics, which may contribute to improved clinical outcomes in NVG management.

**Supplementary Information:**

The online version contains supplementary material available at 10.1186/s13104-025-07313-0.

## Introduction

Glaucoma is the leading cause of irreversible blindness and the second most common cause of blindness globally, resulting in significant vision loss worldwide [1]. Neovascular glaucoma, a secondary form caused by retinal vascular ischemia, is increasingly prevalent due to rising incidents of diabetic retinopathy and retinal vascular accidents, constituting approximately 30% of uncontrolled glaucoma cases [2]. Glaucoma drainage device (GDD) implantations are the most effective and safer options among the surgical treatments for neovascular glaucoma [3]. Managing intraocular pressure (IOP) is crucial to glaucoma treatment [4, 5]. Corneal biomechanics impact both IOP measurement accuracy and the progression of glaucomatous optic nerve damage [6]. Conversely, reducing IOP can alter corneal biomechanics and ocular metrics [7].

Studies using the Ocular Response Analyser revealed that glaucoma surgeries, like trabeculectomy and GDD implantation, can reduce corneal hysteresis and the corneal resistance factor [8, 9]. The reduction in IOP has been associated with decreased axial length and anterior chamber depth (ACD), though these changes vary based on glaucoma type, IOP reduction level, and the surgical approach [10, 11].

While Ahmed Glaucoma Valve (AGV) is mainly treatment for refractory NVG, its biomechanical and biometric effects on anterior segment are poorly understood, limiting personalized postoperative care. Although preliminary evidence suggests that GDD induce anterior segment remodeling [12], no studies have systematically AGV-specific and biomechanical changes in NVG. Understanding these interactions is essential because NVG exhibit unique anatomical vulnerabilities. To address this gap, our study aims to specifically examine the impact of AGV implantation on ocular biometric changes and corneal biomechanics in NVG. Since neovascular glaucoma involves ongoing membrane formation, which might persist post-AGV implantation, the alterations in biometric and biomechanical parameters might differ in this glaucoma type. We focus on analyzing changes in anterior chamber parameters, axial length, refraction, and corneal biomechanics following uneventful AGV implantation.

## Methods

### Subjects and study design

This prospective comparative study evaluates the corneal biomechanics before and after AGV implantation in patients with NVG. The study enrolled 18 eyes from 18 patients diagnosed with NVG. The study was conducted at Khalili Hospital in Shiraz, Iran, between January 2023 and January 2024. All participants provided informed consent before their inclusion in the study, and ethical approval was obtained from the Shiraz University of Medical Sciences ethics committee.

#### Inclusion/Exclusion criteria

Inclusion criteria encompassed NVG patients with uncontrolled IOP despite maximum medication or those with neovascular glaucoma and peripheral anterior synechia suitable for AGV. Exclusion criteria involved corneal opacity, previous keratoplasty, anterior staphyloma, previous ocular trauma, prior corneal refractive surgery and scleral band/buckling surgeries. Complications during AGV surgery led to patient exclusion, emphasizing the primary goal: analyzing pre- and post-operative parameter changes for nuanced insights into AGV's effects on ocular dynamics, conducted at baseline and three months post-surgery.

#### Patients evaluation

For all the study participants, we recorded demographic details, including age, sex, highest baseline IOP, central corneal thickness (CCT), topical hypotensive medications, history of previous ocular surgeries, and history of drug allergy at baseline. Preoperative assessments were conducted to establish baseline values for corneal biomechanical properties and corneal biometery. These assessments were performed using the Corvis ST (Oculus, Germany) to measure parameters such as corneal deformation amplitude, applanation times, applanation lengths, applanation velocities, and peak distance. The comprehensive evaluation encompassed various ocular parameters such as anterior chamber metrics (axial length, Keratometric values, ACD) assessed using the IOL Master 700(ZEISS, Germany). These assessments were repeated after 3 months postoperatively. The main outcome of the present study was to evaluate the changes in the corneal biomechanical and biometrical values before and 3 months after AGV implantation. So, this study aimed to evaluate post-AGV biometry and biomechanical changes as a first-step exploratory analysis, given the scarcity of such data in NVG eyes. We developed a structured questionnaire for our study(supplementary material).

### Surgical technique

All patients underwent a meticulous preoperative regimen involving the administration of a 10% iodine/povidone solution and the placing a lid speculum. This was followed by a precise injection of bevacizumab (0.625 mg; 0.025 ml of StivantR, CinnaGen Co., Iran) 4 mm posterior to the limbus using a 30-gauge needle—the bevacizumab injection aimed to manage neovascularization and minimize bleeding during the subsequent AGV implantation. All Surgeries were conducted by a single glaucoma specialists A.A., utilizing the FP7 AGV implant from New World Medical Inc., CA, USA.

The surgical sequence commenced with anchoring a corneal traction suture with 7–0 silk, followed by a limbal-based peritomy in the supratemporal quadrant. The implant plate was precisely positioned 10–12 mm posterior to the limbus and secured using 7–0 silk sutures for stability.

Further in the procedure, a precise corneal stab incision was made to facilitate the introduction of viscoelastic material. A trimmed silicone tube was delicately inserted through a paracentesis into the anterior chamber using a 23-gauge needle 2 mm from the limbus. This tube was carefully sutured to the sclera using 10–0 nylon and safeguarded by a full-thickness donor scleral patch graft. The surgery concluded with meticulous closure of the conjunctiva, employing 8–0 Vicryl sutures to ensure comprehensive surgical precision and promote optimal post-operative healing.

Postoperative care and follow up: Topical ciprofloxacin 0.3% prescribed for all eyes and discontinued one week postoperatively. Topical betamethasone 0.1% were discontinued in a tapering fashion for 4 to 6 weeks after surgery. No additional anti-VEGF injections were administered postoperatively and any patients with recurrent neovascularization who needed anti-VEGF injections were excluded from the study. All patients were visited one day, one week and three months after surgery.

### Statistical analysis

Statistical analysis was conducted using SPSS for Windows (version 22, SPSS Inc., Chicago, IL, USA). Continuous variables were assessed for normality using the Shapiro–Wilk test and visual inspection of Q-Q plots. Data with non-normal distribution (p < 0.05) were analyzed using the non-parametric Wilcoxon signed-rank test, while normally distributed variables were compared with paired t-tests. Categorical data were evaluated with Chi-square/Fisher’s exact tests. Independent variables were: baseline biometrical and biomechanical parameters and dependent variables were post-op changes of these values. The association between intraocular pressure (IOP) at the 3-month follow-up and axial length (AL), anterior chamber depth (ACD), central corneal thickness (CCT), deformation amplitude (DEF AMP), and keratometry was assessed using Spearman's rank correlation coefficient. Statistical significance was set at a threshold of p < 0.05.

## Result

A total of 20 subjects were selected based on our inclusion criteria. Of these, two subjects didn’t complete the imaging work-ups that should have been done for the assessment, so 18 subjects (response rate: 90%) participated in this study. Table 1 demonstrates the baseline characteristics of the study population.

**Table 1:** clinical characteristics of 18 eyes of 18 subjects

Parameters	Number = 18	Percentage %
Mean age; year (SD)	57.44 ± 10.325	
Sex		
Male	10	55.6
Female	8	44.4
Lens status		
Phakic	6	33.3
Pseudophakia	12	66.7
Cup-to-disk ratio		
30	1	5.6
70	4	22.2
80	1	5.6
90	1	5.6
100	11	61.1

SD: standard deviation

The mean preoperative IOP was 36.65(± 12.3) mmHg, which was significantly reduced to 15.5(± 2.64) mmHg post-operatively (p < 0.001). Table 2 presents the mean values of ACD, AL, K1, K2, CCT, Applanation1 length, Applanation 2 length, Applanation 1 velocity, Applanation 2 velocity, Peak distance, and Deformation amplitude preoperatively and three months after surgery. Among these variables, AL, K1, K2, and CCT significantly decreased (p < 0.001). ACD showed an increase in amount postoperatively, which was significant (p = 0.037). The Peak distance and Deformation amplitude increase were also significant (p < 0.004, < 0.001, respectively). Our study showed no significant change in preoperative measures of applanation length 1, applanation length 2, applanation velocity 1, and applanation velocity 2 with the amount of these variables postoperatively.

**Table 2:** Comparison of preoperative and postoperative IOP, biometric, and keratometry parameters of 18 eyes of 18 subjects

Parameters	Preoperative mean ± SD	3 months postoperative mean ± SD	P-value
IOP with Corvis (mmHg)	36.65 ± 12.30	15.50 ± 2.64	< 0.001
ACD(mm)	3.85 ± 0.78	3.91 ± 0.77	0.037
AL(mm)	23.42 ± 0.76	23.21 ± 0.76	< 0.001
K1(diopter)	43.94 ± 1.71	43.44 ± 1.64	< 0.001
K2(diopter)	45.51 ± 2.06	44.72 ± 2.02	< 0.001
CCT(mm)	570.06 ± 29.43	531.94 ± 30.53	< 0.001
Applanation 1 length (mm)	2.34 ± 0.30	2.25 ± 0.32	0.082
Applanation 2 length (mm)	2.28 ± 0.37	2.19 ± 0.43	0.240
Applanation 1 velocity (m/s)	0.14 ± 0.19	0.11 ± 0.03	0.227
Applanation 2 velocity (m/s)	− 0.15 ± 0.07	− 0.19 ± 0.13	0.348
Peak distance (mm)	3.33 ± 0.72	3.66 ± 0.78	0.004
Deformation amplitude (mm)	0.58 ± 0.18	0.72 ± 0.22	0.001

IOP: intraocular pressure; ACD: anterior chamber depth; AL: axial length; K1: flat keratometry; K2: steep keratometry; CCT: central corneal thickness

**Fig. 1:**
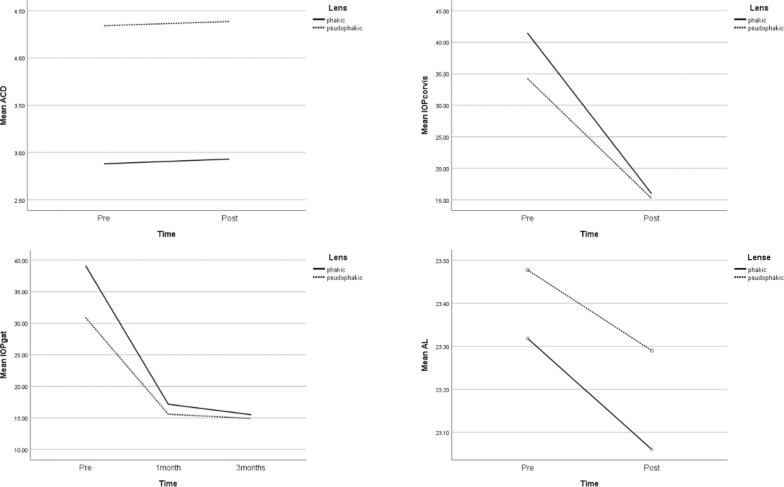
Comparison of preoperative and postoperative means of ACD, IOP (measured by Corvis and GAT) and AL during time and also based on lens status

**Fig. 2:**
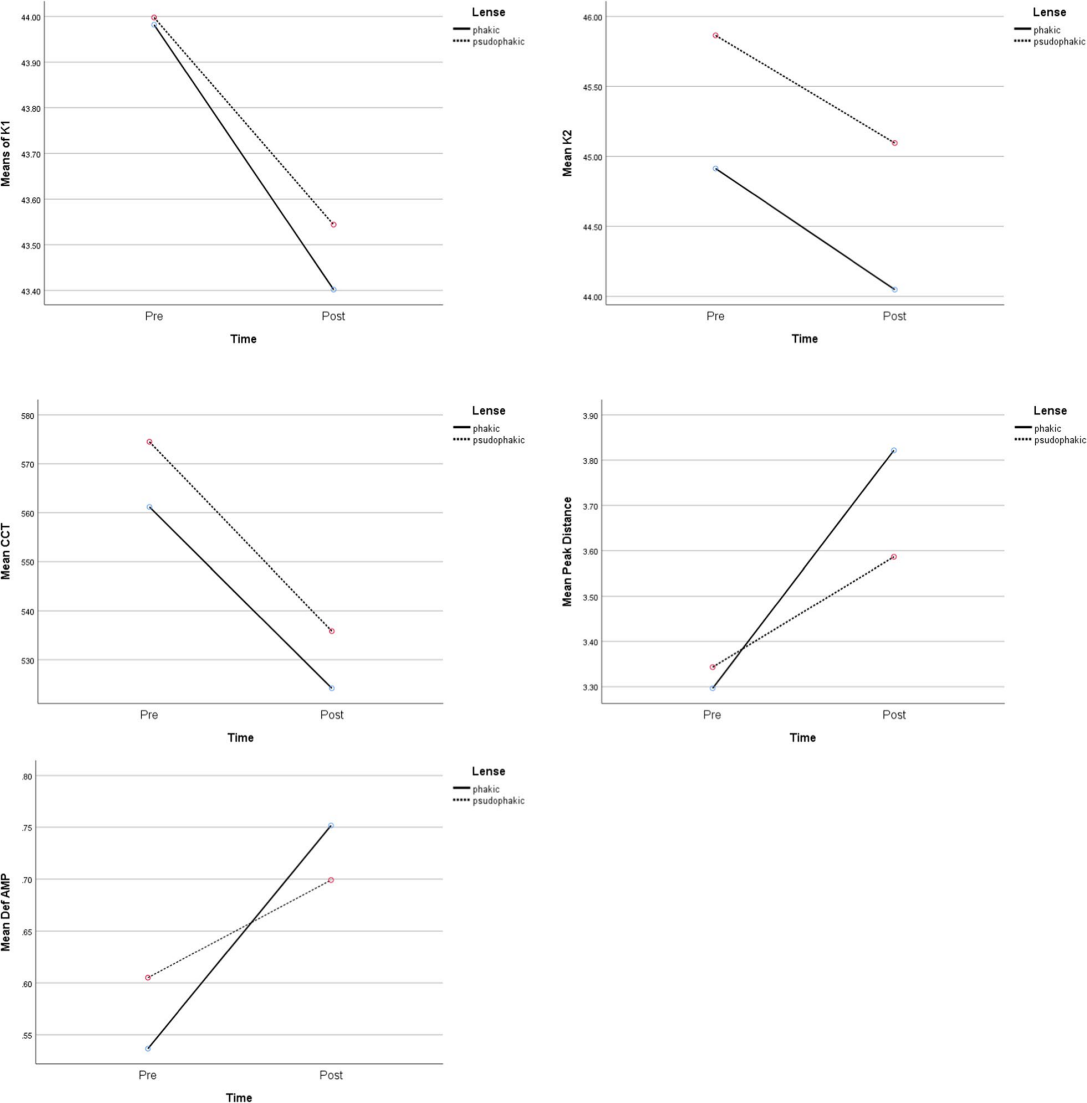
Comparison of preoperative and postoperative means of K1, K2, CCT, Peak distance, Deformation amplitude during time and also based on lens status

Table 3 presents the results of repeated measure analysis of variants between ACD, IOP with Corvis, IOP with GAT IOP, AL, K1, K2, CCT, Peak distance, deformation amplitude, Time, Lens status, and Interaction between time and lens status. All indices changed significantly during this period in this study, which was after three months of follow-up. An increasing pattern was seen in ACD, Peak distance, and Deformation amplitude, while other parameters showed a decreasing pattern. There was no significant change after AGV implantation based on lens status, which was divided into two groups of phakic and pseudophakia, except for mean-ACD. Mean ACD was greater in the pseudophakia lens than the phakic lens (p-value: < 0.001). According to the last column, which shows the interaction between time and lens status, none of the indices changed significantly. In other words, the pattern of changes from preoperative to postoperative examination three months after surgery was similar in either the phakic or pseudophakic lens group (Figs. 1 and 2).

**Table 3:** Comparison of preoperative and postoperative IOP, biometric, and keratometry parameters of 18 eyes of 18 subjects during the time, based on their lens status and also the interaction between time and lens

Parameters	Phakic		Pseudophakic		P-value		
	Preoperative	Postoperative	Preoperative	Postoperative	Time	Lens	T × L*
ACD	2.88 ± 0.41	2.93 ± 0.33	4.34 ± 0.30	4.38 ± 0.29	0.042	< 0.001	0.813
IOP with Corvis	41.47 ± 16.78	16.00 ± 3.35	34.24 ± 9.31	15.25 ± 2.34	0.004	0.504	0.635
IOP with GAT	39.17 ± 13.27	15.50 ± 3.39	30.92 ± 7.46	14.92 ± 2.46	0.001	0.25	0.523
AL	23.32 ± 0.70	23.06 ± 0.65	23.47 ± 0.82	23.30 ± 0.83	< 0.001	0.624	0.093
K1	43.98 ± 2.18	43.40 ± 2.03	43.91 ± 1.53	43.46 ± 1.51	0.001	0.490	0.490
K2	44.91 ± 2.25	44.04 ± 2.19	45.81 ± 1.99	45.06 ± 1.94	0.001	0.284	0.782
CCT	561.17 ± 13.04	524.17 ± 10.51	574.50 ± 34.60	535.83 ± 36.61	< 0.001	0.408	0.818
Peak distance	3.29 ± 0.48	3.82 ± 0.84	3.34 ± 0.84	3.60 ± 0.78	0.001	0.635	0.275
Deformation amplitude	0.54 ± 0.16	0.75 ± 0.22	0.60 ± 0.19	0.70 ± 0.22	< 0.001	0.953	0.072

ACD: anterior chamber depth, IOP: intraocular pressure; AL: axial length; K1: flat keratometry; K2: steep keratometry; CCT: central corneal thickness

^*^: Interaction effect between Time and Lens

Repeated measure analysis of variants was performed. Based on this analysis, age had a negative effect on IOP, which was measured with Corvis pre-operatively and statistically significant. (β_age_ = − 0.063) (p = 0.019). To illustrate, older patients had less IOP at the baseline. However, postoperatively, the effect of age was not statistically significant. (β_age_ = − 0.05). The interaction between time and age was not significant either. Analysis for IOP, which was measured with GAT, showed similar results. In particular, age negatively affected IOP preoperatively, which was statistically significant (β_age_ = − 0.526) (p = 0.048), however, postoperatively, results did not bring significant changes (β_age(3 months)_ = 0.008). The interaction between time and age was not significant either (Table 4).

**Table 4:** Comparison of preoperative and postoperative IOP of 18 eyes of 18 subjects during time, based on age, and interaction between time and age

Parameters	Phakic		Pseudophakic		P-value		
	Preoperative	Postoperative	Preoperative	Postoperative	Time	Age	T × A^a^
IOP with Corvis	41.47 ± 16.78	16.00 ± 3.35	34.24 ± 9.31	15.25 ± 2.34	0.004	0.019	0.056

		One month	Three months		One month	Three months			
IOP with GAT	39.17 ± 13.27	17.17 ± 5.49	15.50 ± 3.39	30.92 ± 7.46	15.58 ± 2.81	14.92 ± 2.46	0.001	0.048	0.033

^a^Interaction effect between time and age

**Fig. 3:**
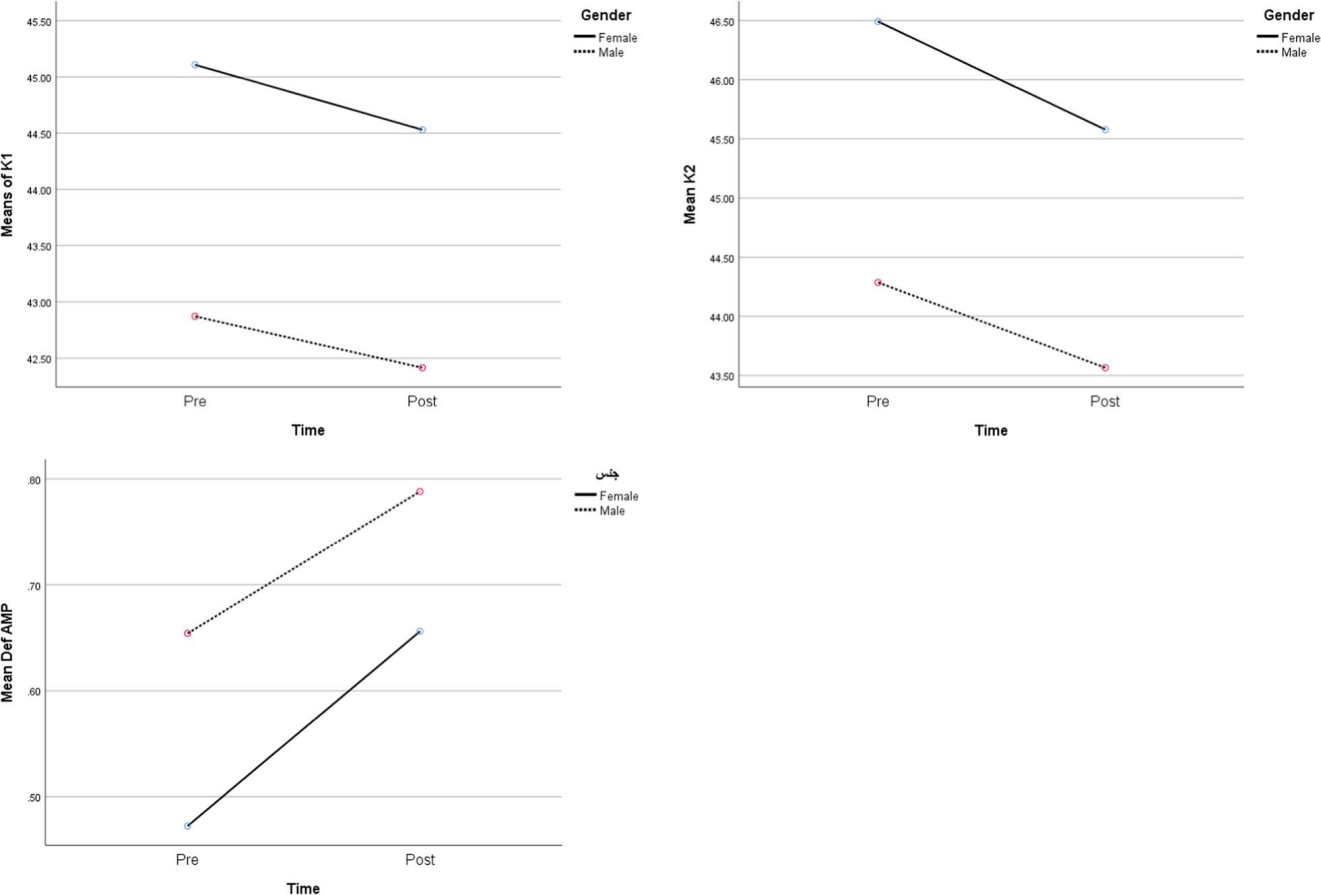
Comparison of preoperative and postoperative means of K1, K2, CCT, Deformation amplitude during time and also based on gender

Figure 3 shows the effect of sex on mean-K1, mean-K2, and mean-deformation amplitude, respectively. Although mean-K1 and mean-K2 were greater in women in comparison to men, with p-values of 0.008 and 0.033, which indicates the significance, this pattern was different in mean-Deformation amplitude, which showed greater values in men than in women, with a statistically significant p-value of 0.037. In contrast, the interaction between time and sex did not show significant changes in any groups. (p-value k1 = 0.504) (p-value K2 = 0.577) (p-value Deformation amplitude = 0.30).

Table 5 presents the correlation between postoperative IOP (at month 3) with biomechanical and keratometry features of 18 eyes of 18 subjects; none of the correlations brought significant changes.

**Table 5:** The correlation between postoperative IOP(in the first month) with biomechanical and keratometry features of 18 eyes of 18 subjects

Clinical characteristics*	Correlation coefficient	P-value
IOP with Corvis vs ACD	− 0.142	0.575
IOP with Corvis vs AL	− 0.171	0.496
IOP with Corvis vs K1	0.154	0.541
IOP with Corvis vs K2	− 0.126	0.618
IOP with Corvis vs CCT	− 0.089	0.726
IOP with Corvis vs. Applanation 1 Length	0.317	0.199
IOP with Corvis vs. Applanation 2 Length	− 0.138	0.584
IOP with Corvis vs. Applanation 1 Velocity	− 0.189	0.453
IOP with Corvis vs. Applanation 2 Velocity	0.250	0.316
IOP with Corvis vs. Peak Distance	− 0.136	0.590
IOP with Corvis vs DEF AMP	− 0.191	0.447

IOP: intraocular pressure; ACD: anterior chamber depth, Al: axial length; K1: flat keratometry; K2: steep keratometry; CCT: central corneal thickness; DEF AMP: deformation amplitude

## Discussion

To our knowledge, this is the first study to report corneal biomechanical and biometrical changes after neovascular glaucoma treatment by AGV implantation.

In our investigation focusing on GDD implantation for neovascular glaucoma (NVG) and patients with neovascular iris (NVIs), we observed a decrease in axial length (AL). This finding aligns with the results reported by Miraftabi et al. [12] and is consistent with numerous studies that explored the outcomes of trabeculectomy [13, 14]. Bae et al. reported an AL reduction of 0.28 mm as measured by the IOL Master [15]. Remarkably, our study observed a comparable decrease in AL, with a reduction of 0.21 mm.

Additionally, our study highlighted a correlation between the reduction in IOP following surgical and pharmacological interventions and a concurrent decrease in AL.

The significantly deeper ACD in pseudophakic eyes likely reflects: (a) anatomical changes from lens extraction, (b) reduced iris-lens contact in these eyes with prior complex surgeries. Postoperatively, a common occurrence in trabeculectomy and AGV implantation is a shallow anterior chamber, usually resolving within two weeks [16, 17]. Our study reveals a prolonged increase in ACD, particularly notable in pseudophakic individuals. This observation contradicts the findings by Miraftabi et al. [12] and Ana Marta et al. [18]. Hunkyu Seo et al. Postulated that vitreous pressure is more than anterior chamber pressure [19], so our finding difference may arise from the fact that our patient had higher IOP than the Miraftab study, which caused more fluid accumulation in the vitreous and subsequent anterior lens iris diaphragm push.

In our study, we observed a decrease in both steep and flat corneal curvature and a reduction in astigmatism, postoperatively. This contradicts numerous studies that reported significant induction of with-the-rule (WTR) astigmatism changes after trabeculectomy related to corneal and scleral sutures [11, 19]. In contrast to the other studies that used GDD for controlling IOP [12, 18], our study showed that flat and steep keratometry decreased after GDD implantation. Furthermore, we noted a substantial decline in central corneal thickness (CCT), attributed to the reduction in corneal edema resulting from the lowered IOP that is the same as Na Wu et al. that shows a decrease in CCT after treatment of POAG with prostaglandins analog [20]. However, Miraftab et al. showed no change in CCT after GDD implantation [12]. However, as mentioned earlier, our patients had greater levels of IOP, so the changes in the CCT are more pronounced. So, the observed 0.5–0.8 D flattening in keratometery likely results from: (1) AGV plate-induced posterior scleral displacement (ΔAL), (2) corneal dehydration (CCT reduction), and (3) remodeling of corneoscleral collagen (supported by hysteresis changes). The temporal pattern suggests biomechanical effects dominate early.

Our investigation showed that despite a decrease in CCT postoperatively, there is no difference in AL 1 and AL2 before and after the surgery, in contrast to a study carried out by Bruno Freitas Valbon et al. that showed a positive correlation between CCT and AL 1 in the normal population [21]. This finding may be due to a rise in IOP in NVG patients in a short time and dysregulation in corneal water hemostasis that causes corneal edema and an increase in central corneal thickness. This abrupt corneal change may not affect all corneal biomechanics.On the other hand,our study is in line with the results of Vincent Borderie et al. which noted that AL 1 and AL2 and applanation velocity 1 are not correlated with IOP [22].

We also showed that despite a decrease in central corneal thickness after GDD implantation, there was no change in applanation velocities 1 and 2. This finding is contrary to the study carried out by Bruno Freitas Valbon, which showed that applanation velocity has a negative correlation with central corneal thickness in normal populations [21], and Shuichiro Aoki, who showed an increase in applanation velocity 1 after POAG treatment [22]. Our Contradictory finding may be due to high intraocular pressure in NVG patients that dysregulates water hemostasis in the cornea.

Our study also demonstrates that deformation amplitude increases after GDD implantation; this finding is aligned with Shuichiro Aoki et al. who noted that deformation amplitude increases after treatment in POAG patients [22]. Lisa Ramm et al. showed more deformation amplitude in normal-pressure glaucoma patients versus high-pressure glaucoma patients (POAG is a classification on its own) [23]. Our finding may be due to high IOP and thicker CCT before GDD implantation with subsequent stiffening of the cornea. After GDD implantation and significant decrease in IOP and CCT, the cornea becomes more deformable and deformation amplitude increases.

Our research also highlighted that peak distance increases after GDD implantation. This finding is in agreement with other studies showing a low peak distance correlated with stiff corneas [24, 25]. In NVG, due to high IOP, the peak distance is low before IOP lowering, and will increase after AGV implantation and IOP reduction.

*Future Directions*: Recent advances in computational ophthalmology [24] demonstrate how deep learning can predict postoperative outcomes from preoperative biometry. Applying such methods—particularly temporal graph neural networks [25] or multimodal fusion approaches [26]—could help: (1) identify hidden patterns in our biomechanical datasets, (2) predict which eyes will develop extreme flattening (K > 1D shift), and (3) optimize AGV positioning using finite-element modeling [27]. Future studies could train models on our dataset (publicly available upon request) combined with longer-term follow-up data.

## Limitations

The 3-month follow-up period in this study limits our ability to assess long-term stability of biomechanical changes or late-onset complications. While this timeframe captures the immediate postoperative period when most biometry changes occur, future studies with 6–12-month follow-up are needed to evaluate whether these changes persist, progress, or regress over time. On the other hand, this study’s single-arm design precludes definitive attribution of observed changes solely to AGV implantation, as natural disease progression or adjunct therapies (e.g., anti-VEGF injections) may contribute. While randomized controlled trials are ideal, our cohort reflects real-world NVG management where AGV is often employed alongside adjuvant treatments. Future studies with matched controls or propensity-score analyses would help delineate AGV-specific effects.

## Conclusion

Treatment of NVG patients with AGV implantation can change the axial length and even keratometric measures. The acute IOP change can affect corneal biomechanical indices that are related to IOP (deformation amplitude, peak distance, and CCT). On the other hand, acute central corneal thickness change due to water dysregulation does not affect those corneal biomechanical indices that purely evaluate the cornea and exclude IOP (AL1, AL2, and applanation velocity 1 and applanation velocity 2). Our short-term observations suggest that AGV implantation induces measurable changes in ocular biometry and corneal biomechanics during the first postoperative months. Further investigations with larger cohorts and longer follow-up periods are warranted to better understand how acute IOP changes impact corneal biomechanics, which could ultimately refine postoperative monitoring strategies, optimize refractive management, and enhance surgical outcomes for neovascular glaucoma patients treated with AGV implantation.

## Supplementary Information

Supplementary Material 1.

## Data Availability

The datasets used and/or analyzed during the current study are available from the corresponding author upon reasonable request.
