# Supplementary material for: Short-term effects of Ahmed valve implantation on ocular biometry and corneal biomechanics in neovascular glaucoma
Source: BMC Res Notes. 2025 Jul 1;18:256. doi: 10.1186/s13104-025-07313-0 (PMC12217899; doi:10.1186/s13104-025-07313-0)
Supplement: Supplementary file 1 — Supplementary Material 1. [file 13104_2025_7313_MOESM1_ESM.docx]

**Data Gathering Form**

**Title:** Short-term Effects of Ahmed Valve Implantation on Ocular Biometry and Corneal Biomechanics in Neovascular Glaucoma

PATIENT IDENTIFIER: ___________
Date of Surgery: ___ / ___ / ___
Eye (OD/OS): ___

BASELINE DATA (Pre-operative)

| Parameter | Value | Unit | Device Used |
| --- | --- | --- | --- |
| Age |  | Years |  |
| Sex(M/F) |  |  |  |
| NVG Cause |  |  |  |
| IOP |  | mmHg | Goldmann |
| Visual Acuity(LogMAR) |  |  | Snellen |
| Axial Length |  | mm | IOL master 700 |
| Anterior chamber depth |  | mm | Pentacam |
| Keratometery |  | diopter | Pentacam |
| Corneal Applanation Time |  | s | Corvis ST |
| Corneal Applanation velocity |  | m/s | Corvis ST |
| Deformation Amplitude |  | mm | Corvis ST |
| Peak distance |  | mm | Corvis ST |
| Central Corneal thickness |  | micron | Corvis ST |

Follow Up Data(Post-operative)

| Parameter | Value | Unit | Device Used |
| --- | --- | --- | --- |
| Age |  | Years |  |
| Sex(M/F) |  |  |  |
| NVG Cause |  |  |  |
| IOP |  | mmHg | Goldmann |
| Visual Acuity(LogMAR) |  |  | Snellen |
| Axial Length |  | mm | IOL master 700 |
| Anterior chamber depth |  | mm | Pentacam |
| Keratometery |  | diopter | Pentacam |
| Corneal Applanation Time |  | s | Corvis ST |
| Corneal Applanation velocity |  | m/s | Corvis ST |
| Deformation Amplitude |  | mm | Corvis ST |
| Peak distance |  | mm | Corvis ST |
| Central Corneal thickness |  | micron | Corvis ST |
